# Preparing for pharmacy-based delivery of long-acting injectable antiretrovirals: a pre-implementation study

**DOI:** 10.1186/s12913-025-12971-8

**Published:** 2025-06-05

**Authors:** Jennifer Cocohoba, Yvette Cuca, Elizabeth Sherman, Kelly Hester, George Udeani, Michael Sigua, Parya Saberi

**Affiliations:** 1 Department of Clinical Pharmacy, University of California San Francisco School of Pharmacy, 521 Parnassus Avenue, C-152, Box 0622, San Francisco, CA 94143 USA; 2 Department of Community Health Systems, University of California San Francisco School of Nursing, 490 Illinois Street, Floor, Box 0608, San Francisco, CA 94143 USA; 3 Department of Pharmacy Practice, Nova Southeastern University, Barry and Judy Silverman College of Pharmacy, 3200 South University Drive, Fort Lauderdale, FL 33328 USA; 4 Division of Infectious Disease, Memorial Healthcare System, Hollywood, FL USA; 5 Department of Pharmacy Practice, Auburn University Harrison College of Pharmacy, Walker Bldg, 2316, 362 Thach Concourse, Auburn, AL 36849 USA; 6 Departments of Pharmacy Practice and Translational Medical Sciences, Texas A&M University, Schools of Pharmacy and Medicine, 1010 W Ave B, Kingsville, TX USA; 7 Division of Prevention Science, University of California San Francisco School of Medicine, 550 16th St 3rd Floor, San Francisco, CA 94158 USA

**Keywords:** Pharmacy, Long-acting injectable antiretrovirals, HIV, Antiretroviral therapy, Implementation science, Pre-implementation study

## Abstract

**Background:**

Use of long-acting injectable antiretroviral therapy (LA-ART) for human immunodeficiency virus (HIV) treatment and prevention is increasing, but there are challenges that could limit broad expansion of these important treatments. The goal of this study was to explore attitudes, barriers, and facilitators for implementing LA-ART administration within community pharmacies.

**Methods:**

We conducted a mixed-methods study focusing on pre-implementation aspects of community pharmacy-administered LA-ART. Pharmacists, clinic staff members, and persons with HIV completed a baseline survey followed by an individual semi-structured qualitative interview with questions based on the Consolidated Framework for Implementation Research (CFIR v.1.0).

**Results:**

A total of 63 participants (pharmacist *n* = 19, clinic staff *n* = 20, person with HIV *n* = 24) from Alabama, California, Florida, and Texas were included in the study. Most pharmacist participants were employed in retail pharmacies that processed fewer than 500 prescriptions per day (75%) and most had more than 10 years of experience working with people with HIV (53%). Clinic staff participants were also highly experienced in working with people with HIV (55%). People with HIV enrolled in the study were either on oral antiretroviral therapy but interested in LA-ART (59%) or were already on LA-ART (29%). Clinics were the preferred location for LA-ART administration while community pharmacy was the second preferred location. Attitudes regarding pharmacy-based administration of LA-ART were mostly positive, with the primary facilitator being positive experiences or established relationships between clinics and pharmacies or persons with HIV and their pharmacies. Barriers included concerns about pharmacy staffing, training, space, privacy, and pharmacy reimbursement for services.

**Conclusion:**

Involving community pharmacies in the administration of LA-ART could expand access to HIV treatment and prevention medications. Outlining best practices that leverage facilitators and overcome barriers can help clinics, pharmacy, and communities expand this novel model of care.

**Trial registration:**

ClinicalTrials.Gov Registration: NCT05152953 (posted 12/10/2021).

**Supplementary Information:**

The online version contains supplementary material available at 10.1186/s12913-025-12971-8.

## Introduction

Long-acting injectable antiretroviral therapy (LA-ART) offers an alternative option for human immunodeficiency virus (HIV) treatment and prevention. These novel treatments arrive at a time when the U.S. Department of Health and Human Services predicts a 5% shortage in HIV clinician workforce and a simultaneous 14–36% increase in service demands [1]. Currently, there are intramuscularly administered (IM) as well as subcutaneously administered (SC) LA-ART available and most are administered within clinics by nurses. An implementation study of cabotegravir LA with rilpivirine LA found that clinic-based administration increased in efficiency over time, yet there are existing concerns that incorporating this important intervention into some clinics may still increase healthcare system and organizational stress [2, 3]. Clinics may need increased staffing to administer injections and navigate insurance benefits. Pressures to keep clinic-administered medication budgets low may negatively influence the real-world provision of LA-ART. Patients may face barriers securing clinic appointments for injections, may have to navigate around clinic business hours, secure transportation, or travel long distances to maintain LA-ART adherence [4]. The potential increased pressures on patients and health systems renders it imperative to explore alternative delivery strategies for LA-ART.

There are approximately 67,000 community pharmacies in the United States [5]. Forty-four states allow pharmacists to administer either IM or SC injectable medications other than vaccines, though some require collaborative practice protocols or limit administration to specific medications [6]. Pharmacists have demonstrated their importance as HIV care team members who support treatment adherence and improve virologic outcomes [7–9]. This unique combination of an abundance of pharmacy locations, accessibility within the community, and prior evidence of pharmacist impact on HIV treatment outcomes makes pharmacies ideal alternative delivery sites.

It is assumed that pharmacies will participate in administering LA-ART, yet there are several unknowns. To date, there have been limited assessments of pharmacist or healthcare practitioner attitudes towards pharmacist administration of LA-ART [10, 11]. Financial and human resources, pharmacist training, or envisioned changes in workflow have not been outlined. Lastly, little is known about patient acceptability of LA-ART administration in pharmacies. Without these critical pieces of information, successful implementation will be limited to specific, well-resourced pharmacies that few patients will be able to access. Supporting community pharmacies to serve as administration sites can help expand and ensure consistent patient access to LA-ART. The goal of this study is to explore various aspects of LA-ART administration within community pharmacies by developing an in-depth understanding of the attitudes, barriers, and facilitators for implementing this intervention.

## Methods

### Study design, recruitment, and eligibility

We conducted a convergent-parallel mixed-methods study that focused on pre-implementation aspects of community pharmacy-administered LA-ART [12]. This design was selected to form both a breadth and depth of understanding of the factors that could influence the development of this novel pharmacy practice. Participants were recruited from four geographic areas: Central Alabama, Northern California, Southern Florida, and Eastern Texas. Eligible individuals were required to be aged 18 years or older and have access to a telephone or the internet to complete the study activities. These are locales with high HIV prevalence, diverse populations, differences in pharmacist scope of practice, and variations in clinic resources. As there was no specific hypothesis being tested in this study, a formal sample size calculation was not conducted, but data adequacy was considered when estimating recruitment targets [13]. Purposive and maximum variation sampling methods were employed.

Three key stakeholder groups were recruited including pharmacy staff members, persons with HIV, and clinic staff members. Pharmacy staff members had to be employed at a community or outpatient health system pharmacy that served persons with HIV as at least 5% of their population. Persons with HIV were required to have a self-reported diagnosis of HIV and be currently taking antiretroviral therapy. Clinic staff were required to have been employed at the time of the study in a clinic practice where people living with HIV comprised at least 5% of the clinic population. Prior studies have described implementation of LA-ART programs in clinics; in these papers and in our own research team’s experience, multiple team members often participate in the day-to-day execution of these programs. Our study similarly wished to include perspectives from a variety of clinic disciplines potentially involved in scheduling, adherence support, clinical evaluation, as well as administration of LA-ART. Of note, ambulatory care or HIV pharmacist specialists practicing within clinics were included in the clinic stakeholder group because their duties may be expanded beyond those of community pharmacy staff via collaborative practice protocols, they may not dispense medications, and in prior LA-ART studies they were often a key member of clinic implementation teams.

Recruitment occurred via a variety of methods depending on the stakeholder group and location. To recruit patient-participants, flyers were physically or electronically distributed to regional clinics and pharmacies known to site investigators. Recruitment emails were sent directly to pharmacists and clinic staff who were part of the site investigators’ professional networks. A $50 gift card was provided to patient-participants who completed the study. A $100 gift card was provided to clinic or pharmacy staff who completed the study due to the lengthier nature of the interview and market value for services. The research was approved by WCG Institutional Review Board through a reliance agreement with the University of California San Francisco, and was reviewed by Auburn University Institutional Review Board, Texas A&M University Institutional Review Board, and Nova Southeastern University Institutional Review Board.

### Exploration of LA-ART

At the time the research study was planned, there was only one LA-ART product commercially available: cabotegravir LA with rilpivirine LA used for HIV treatment. Over the course of the study other LA-ART were approved by the FDA including cabotegravir LA for HIV prevention and lenacapavir SC for HIV treatment. Survey questions and interview guides were designed intentionally broad, using general wording such as “long-acting injectable antiretroviral medications” included in interview prompts and survey questions. This allowed the opportunity for participants to discuss their overall perceptions of LA-ART, although most participants were only aware of the products administered intramuscularly.

### Survey questions

Participants filled out an electronic survey (REDcap, Vanderbilt University) at baseline to collect demographic characteristics such as age, sex at birth, gender identity, ethnicity, race, highest educational attainment, current employment, and income category. All health care providers were asked about the length of time in practice, length of time in current place of employment (serving patients with HIV), role/title, and estimated proportion of total patients with HIV served at their location. The pharmacy staff were also asked what type of pharmacy they worked in. Persons with HIV were asked about length of time since HIV diagnosis, treatment regimen, type of pharmacy used, and how often they physically visit the pharmacy. Persons with HIV also filled out the Trust in Physician Scale, which assessed their opinion on 11 statements with a 5-point Likert scale in which a score of 1 corresponded to “strongly disagree” and a score of 5 corresponded to “strongly agree” [14]. The Trust in Physician Scale was adapted to create a “Trust in Pharmacist” Scale by replacing the word “physician” with “pharmacist”.

All participants were asked to rank their location preference for LA-ART administration (clinic, pharmacy, mobile van, home, other). Associations between location preference and stakeholder group were assessed using Fisher’s Exact test. All participants filled out the Acceptability of Intervention Measure (AIM), Intervention appropriateness measure (IAM), and Feasibility of intervention measure (FIM) [15]. Each scale contained 4 items with responses tallied on a 5-point Likert scale. The minimum score that can be received for each scale is 4, with 20 being the maximum. Higher scores indicate greater acceptability, perceived appropriateness, and perceived feasibility. A standard statement was provided just prior to administration of the AIM, IAM, and FIM to clarify the concept of LA-ART administration in pharmacies. (e.g., *“This set of questions will ask you for your opinions about administration of long-acting injectable antiretroviral therapy in a community pharmacy. Administration means that you would go to the pharmacy and someone there (most likely a pharmacist) would prepare the injection and give it to you. There are no right or wrong answers to these questions*.”). Survey responses were analyzed using descriptive statistics. Means for the summary AIM, IAM, and FIM measures, as well as an aggregate measure of the three scales were compared by stakeholder group and geography using one-way analysis of variance (ANOVA). All quantitative data were analyzed using STATA v15.0 (StataCorp, College Station, TX).

### Individual interviews

Following completion of the baseline survey, all participants were scheduled for an individual interview. A semi-structured guide was created for each stakeholder group with questions based on the Consolidated Framework for Implementation Research (CFIR) [16, 17] and is included as a Supplementary file (see Supplement 1). Audio-only interviews were conducted by two research team members (JC and MS) via Zoom and were transcribed as soon as possible after completion. Transcripts were reviewed by the Principal Investigator within 5 business days of completion.

A thematic analysis was conducted using both inductive and deductive approaches to account for the CFIR constructs probed for in the interview guides, adaptations of CFIR for pharmacy, and constructs for implementation and service outcomes [18]. Three research team members (JC, YC, MS) participated in analysis of qualitative data. The process included familiarization with interview transcript data, indexing and coding, exploring other common themes, and interpretation. A small subset of transcripts was reviewed independently by the team members to develop the initial codebook that would be used for all transcripts. The coding team met a total of 8 times over 4 months and reviewed a total of 13 transcripts (4 patient, 4 pharmacy, 5 clinic; 21% of total) to refine the codebook. Discrepancies in coding were resolved through discussion. Transcripts were uploaded to Dedoose (University of California Los Angeles, Los Angeles, CA) for the final analysis and the final set of codes was applied to all data by two study team members (JC, MS). Data were aggregated across all enrolled participants to provide a generalized description of the feasibility, facilitators and barriers for administering LA-ART in pharmacies.

### Data integration

In this convergent parallel design study, data were integrated through connecting and merging [19]. Connection occurred through sampling, given all participants were asked to complete both the quantitative survey and an individual semi-structured interview. Connection was also employed during design; the interview guide was firste generated based on CFIR constructs then the questions were also mapped to the AIM, IAM, and FIM to understand their relationship. For example, the AIM and IAM scales assess acceptability and appropriateness of administration of LA-ART in pharmacies, and questions in the qualitative interview guides probe participants regarding attitudes and reasons why it may or may not be acceptable or appropriate. Merging occurred during data analysis. Survey and interview data were first analyzed separately, then examined in parallel (merged) to assess for complementarity or discordance. Joint displays were created iteratively and used as an analytic tool for examining the data and forming inferences.

## Results

### Participant characteristics

A total of 81 participants were screened for the study. Seven pharmacy staff members who did not live in the targeted geographic areas or whose pharmacies did not serve persons with HIV were classified as ineligible for the study. Four pharmacy staff members were eligible but did not complete the consent form. Seven persons were eligible, completed the consent and baseline survey, but were lost to follow up for interview (2 pharmacy staff members, 2 persons with HIV, and 3 clinic staff members). Sixty-three participants were included in the final analysis. Demographic characteristics of the included participants are described in Table 1.

**Table 1:** Participant demographics

	Pharmacy Staff (*n* = 19)	Clinic Staff (*n* = 20)	People with HIV (*n* = 24)
Location, *n* (%)			
Alabama	6 (32)	4 (20)	6 (25)
California	5 (26)	5 (25)	10 (42)
Florida	5 (26)	5 (25)	6 (25)
Texas	3 (16)	6 (30)	2 (8)
Age, years, mean (SD)	44 (9.6)	43 (10.2)	53 (13.9)
Female sex at birth, n (%)	12 (64)	14 (65)	12 (50)
Gender n (%)			
Man	7 (37)	6 (30)	11 (46)
Woman	12 (63)	12 (60)	12 (50)
Transgender woman	0 (0)	1 (5)	1 (4)
Other/prefer not to state	0 (0)	1 (5)	0 (0)
Self-reported Race, n (%)			
White	7 (37)	11 (55)	7 (29)
Black	3 (16)	4 (20)	12 (50)
Hispanic or Latino	2 (11)	5 (25)	2 (8)
Asian or Pacific Islander	7 (37)	0 (0)	1 (4)
Other	0 (0)	0 (0)	2 (8)
Educational attainment, n (%)			
Completed high school	0 (0)	0 (0)	5 (21)
Some college	0 (0)	2 (10)	9 (38)
Four -year college or Graduate degree	19 (100)	18 (90)	7 (30)

Healthcare workers’ locations and roles are outlined in Table 2. Pharmacy staff (*n* = 19, unique pharmacies = 18) mainly identified as other or mixed race and as cis-gender women. Most worked at small- or medium-sized (< 500 prescriptions processed per day) retail chain pharmacies and had been employed for over 10 years. Clinic staff spanned many different roles (*n* = 20, unique clinics = 16). Most were newer staff members employed by their organizations for less than 5 years, but the majority had been working with people with HIV for 10 years or more. Less than half of pharmacy and clinic staff participants worked in locations that were administering LA-ART (46%). Three pharmacists worked at pharmacies (*n* = 2) that administered LA-ART. In clinics administering LA-ART (*n* = 11), clinic staff reported that their clinic-based pharmacists spent the most time assisting with LA-ART (55%), followed by nurses (20%). Most healthcare workers felt they had at least some working knowledge about LA-ART.

**Table 2:** Characteristics of clinic and pharmacy staff participants

	Pharmacy staff (*n* = 19)	Clinic Staff (*n* = 20)
Role, n (%)		
Pharmacy Manager	8 (42)	N/A
Staff Pharmacist	11 (58)	N/A
Clinic based pharmacist (no medication dispensing)	N/A	6 (32)
Physician or Nurse Practitioner	N/A	4 (22)
Nurse	N/A	3 (16)
Medical assistant/nursing assistant	N/A	1 (5)
Clinic manager or administrator	N/A	1 (5)
Social worker/case manager	N/A	4 (20)
Time employed by organization, n (%)		
< 5 years	7 (37)	8 (40)
6–10 years	4 (21)	7 (35)
> 10 years	8 (42)	5 (25)
Years working with persons with HIV, n (%)		
5 years or less	6 (32)	6 (30)
6–10 years	3 (16)	3 (15)
> 10 years	10 (53)	11 (55)
Number of prescriptions filled per day (pharmacy) OR Number of persons with HIV cared for per year (clinic), n (%)		
< 100 prescriptions daily or patients/year	(0)	1 (5)
100–300 prescriptions daily or patients/year	8 (40)	3 (16)
301–500 prescriptions daily or patients/year	7 (35)	4 (21)
> 500 prescriptions daily or patients/year	5 (25)	11 (58)
Number of persons with HIV participant provides direct care for on a weekly basis, n (%)		
Less than 25 persons with HIV per week	3 (16)	7 (35)
25–50 persons with HIV per week	9 (47)	9 (45)
51–100 persons with HIV per week	3 (16)	2 (10)
More than 100 persons with HIV per week	4 (21)	2 (10)
Type of pharmacy, n (%)		
Independent	3 (16)	N/A
Retail/chain	9 (47)	N/A
Clinic-or hospital associated pharmacy	7 (37)	N/A
Works at a clinic or community pharmacy that administers LA-ART, n(%)	3 (16)	15 (75)
Number of persons getting LA-ART injected by your org, n (%)		
1–10 persons	0 (0)	6 (40)
11–25 persons	1 (33)	5 (33)
> 25 persons	2 (67)	4 (27)
Self-rated knowledge about LA-ART?, n(%)		
Not knowledgeable	2 (11)	0 (0)
Slight/moderately knowledgeable	11 (58)	11 (55)
Very/extremely knowledgeable	6 (32)	9 (45)

Persons with HIV who enrolled in the study had typically been diagnosed over 10 years ago, were mostly undetectable on antiretroviral therapy, and reported excellent adherence to treatment (Table 3). This reflects a population that would potentially be eligible for (or were already on) LA-ART. Most persons obtained ART from retail chain pharmacies, although most received ART through pharmacy delivery services or mail services. Despite this, 72% of people with HIV continued to physically visit a pharmacy at least once every two months. There were 7 people with HIV included in the study who were currently on LA-ART. Of the remaining 17 persons, 59% stated that they would either “probably” or “definitely” consider using LA-ART.

**Table 3:** Characteristics of persons with HIV (*n* = 24)

	*N*(%)
Years since diagnosis	
1–5 years	1 (4)
6–10 years	5 (21)
> 10 years	18 (75)
Currently on ART	24 (100)
Self-reported undetectable viral load at last lab	23 (96)
Self-reported ART adherence in last 30 days	
Very poor/poor	0 (0)
Fair	1 (4)
Very good/Good	1 (4)
Excellent	22 (92)
Pharmacy type used to obtain ART	
Retail/chain	11 (46)
Independent	1 (4)
Clinic-associated	3 (13)
Mail-order	6 (25)
Hospital	2 (8)
Other	1 (4)
How medicines are usually obtained from the pharmacy	
Pick up at pharmacy	7 (29)
Pharmacy delivery or mail	13 (54)
Both	1 (4)
Other	3 (13)
Average # visits to pharmacy	
1 or more times per month	13 (55)
About every 2 months	4 (17)
About every 3 months	1 (4)
Hardly ever	6 (25)
Currently on long-acting injectable ART	7 (29)
If not on LA-ART, likelihood of considering trying LA-ART in the future	
Definitely will not or probably will not consider trying	2 (12)
Might or might not consider trying	5 (29)
Probably will or definitely will consider trying	10 (59)

Across stakeholder groups, most participants selected clinics as their first choice for where LA-ART should be administered (68%). However, community pharmacies were most frequently selected as the second choice (44%) where LA-ART could be administered. In the subgroup of persons already on LA-ART, the first choice was to receive it in clinic (71%), followed by home (29%). Overall, participants viewed pharmacy-administered LA-ART positively as measured by AIM, IAM, and FIM. There were no statistically significant differences in the summary measures of acceptability (*p* = 0.27), appropriateness (*p* = 0.19), or feasibility (*p* = 0.66) by stakeholder group. There was also no statistically significant difference in the aggregate AIM/IAM/FIM measure by geography (*p* = 0.75) or by stakeholder group (*p*=0.38).

### Acceptability of community pharmacies as a place for LA-ART administration

Acceptability was generally high across stakeholder groups and participants primarily expressed positive or neutral attitudes towards community pharmacies providing LA-ART services (Table 4). Three individuals with HIV did express negative attitudes around having community pharmacies administer LA-ART. These negative attitudes stemmed from a lack of trust or relationships with pharmacy providers.

“I wanted to have the security that the person that is doing what she’s doing is knowledgeable and I feel comfortable with it. So going to a pharmacy where there is a shift and there is a different personnel makes me feel uncomfortable. You want it to be safe. You want it to be comfortable. I don’t think this is something that I will do. I know a few guys that will. I won’t do it.” – Person with HIV on LA-ART, FL

**Table 4:** Acceptability, appropriateness, and feasibility for community pharmacies administering LA-ART

Construct	Quantitative	Qualitative	Inferences
Acceptability	*Acceptability of Intervention Measure [AIM*,* mean (SD)]* Pharmacy staff: 15.2 (5.0) Clinic staff: 16.8 (3.0) Persons with HIV: 14.8 (4.0)	“If we dispense the medication, I mean, we might as well inject it. It’s right there.” – Community pharmacist, AL “Anything that helps patients receive appropriate care and appropriate medications, anything that makes that easier for the patient as long as it’s done in a safe way, I am 100% in favor of.” – Nurse Practitioner, AL “I mean, if the pharmacist is qualified to give me a shot yeah, give me a shot.” – Person with HIV not on LA-ART, CA “[T]o me, it’s just not an option, ever, under any circumstances. Because, like I said, these pharmacists, there may be somebody there today, two months they’re gone, six months they’re gone, one year is all new staff, and who knows what their experience is in giving injections.” – Person with HIV on LA-ART, CA	• Pharmacists believed that administration of LA-ART was within scope of activities they already perform • Experiences with pharmacy-administered vaccines and desire for improved accessibility helped clinic staff accept pharmacy-based administration of LA-ART • Many persons with HIV found pharmacy administration acceptable, but a positive existing relationship with pharmacy staff was needed. A select group of people found pharmacy administration of LA-ART unacceptable due to staff turnover.
Appropriateness	*Intervention Appropriateness Measure [IAM*,* mean (SD)]*: Pharmacy staff: 15.5 (4.8) Clinic staff: 16.8 (3.5) Persons with HIV: 14.5 (3.8)	“It really is being able to showcase what we’re able to do as providers and the ability for us to demonstrate why we’re so essential in the healthcare system.” – Community pharmacist/Manager, TX “The biggest advantage would be there’s pharmacies practically on every corner where you could walk in and the pharmacists are more accessible to do it. Whereas if you go to the doctor’s office, you firstly have to have an appointment. You have to pay certain copays. It just becomes a real nightmare. It’s more difficult to see the doctor than it is to see a pharmacist, because there’s so many more pharmacies around that you can actually get it done at. – Specialty pharmacist, FL “I think the advantages would be I don’t have to store it, I don’t have to handle it, I don’t have to take the time to give the injections, and I think it’s more convenient. As I said, we’re rural, so it would be more convenient for a lot of our patients to be able to get it at their pharmacy.” – Nurse, AL “Oh, it would be totally convenient for me. Because I can go in and pick up my, whatever meds I need, like my other meds and get my shot all at one time. I don’t have to travel across town to get it.” – Person with HIV not on LA-ART, CA	• Pharmacists believed medication administration was within their existing scope of practice and wanted to help increase LA-ART accessibility. • Clinic staff found it appropriate for LA-ART to be administered by pharmacists and also hoped this decentralized administration model might decrease their own workloads. • People with HIV felt it would be advantageous to have pharmacies administer LA-ART for convenience and most believed pharmacists had adequate training.
Feasibility	*Feasibility of Intervention Measure [FIM*,* mean (SD)]*: Pharmacy staff: 14.3 (4.7) Clinic staff: 15.5 (3.9) Persons with HIV: 15.0 (3.0)	“I’m assuming the pharmacist is administering it or is involved, so because of their knowledge about medications, they would be able to better assess when issues arise, like missed doses—they kind of know how to triage it.” – Clinic-based pharmacist, TX “It has to be administered on the gluteus muscle. It can’t be done in any other location, so it does need a little more training than injecting a vaccine and an IM on the deltoid. We definitely need more training in that field.” – Specialty pharmacist, FL “I have full confidence. I mean, pharmacies do all kinds of injections all the time. You know, flu shots, COVID vaccines, all the regular vaccines that people get, whooping cough, pertussis, and all that. I have full confidence that they’d be able to do that.” – Social worker, TX “Like I said, they have people who give shots. What’s the difference of a HIV shot than a COVID or a Monkeypox or a flu shot? …I mean they’re trained to give you a shot.” – Person with HIV not on LA-ART, CA	• Pharmacy staff members who likened LA-ART administration to vaccine administration were optimistic about feasibility. Pharmacy staff in leadership recognized that challenges with ordering, space, reimbursement, staffing, and training would need to be addressed for sustainability. • Clinic staff believed it was feasible for pharmacies to administer LA-ART, but some had questions about feasibility of gluteal injections in pharmacy spaces, and most did not have knowledge about financial implications for pharmacies. • People with HIV held greater belief that pharmacies could administer a gluteal injection, but similar to clinic staff lacked knowledge about pharmacy operations and expressed similar concerns about private spaces and staffing.

### Appropriateness of administering LA-ART within community pharmacies

Pharmacy staff, clinic staff, and patients felt it was appropriate for community pharmacies to administer LA-ART (Table 4). This is reflected in participant beliefs about the potential advantages of having LA-ART administered in pharmacies such as expanding access to LA-ART medications, increasing patient convenience, and lowering costs. Clinic staff saw pharmacy-based administration as a pathway to help manage their workload. Pharmacists echoed these positives and the personal and professional benefits of expanding their scope of practice, but some pharmacists and clinic staff provided cautionary logistical information to ground the discussion.

“Ideally I would say the patient would have an appointment. We’d have some kind of appointment system that the patient would be able to make an appointment.” – Community pharmacist/Manager, FL

“I think a specialty pharmacy is well-suited to that. Chain pharmacies that essentially operate more like a retail space [with] a pharmacy in the back, there would really have to be more of a shift … where [a patient] really feel[s] like they’re leaving the retail environment and the long lines and the chaos and going into more of a private clinical space.” – Physician, CA

### Feasibility for community pharmacies to administer LA-ART

There was no difference in stakeholder measures assessing feasibility for community pharmacies to administer LA-ART, though the pharmacy staff group score was slightly lower than that for the other groups (Table 4). Facilitators of feasibility included a general positive regard for pharmacists and prior experiences with pharmacy-administered immunizations. One of the most important factors influencing the potential feasibility of implementation of pharmacy-administered LA-ART was the relationships that both persons with HIV and clinic staff held with pharmacists or their staff. Positive relationships and positive prior experiences were reinforcing facilitators, but it was also understood that these may be unique bonds specific to a person and a pharmacy.

“I think patients are more likely to talk to those pharmacists about things like side effects, adherence issues, challenges with staying in care than they will their provider. Often they’re gonna see their pharmacist more than they see their doctor. I think that there is that kind of closeness that happens with the pharmacist and with the pharmacy team that doesn’t necessarily happen with clinical staff.” – Social worker, TX

“[O]ur physicians have a very good relationship with the pharmacy across the street, but we’ve had a lot of difficulties with certain pharmacies. [It] would be important to see if that relationship was there because they would have to kind of be trusting that the monitoring is occurring, the follow-ups …are occurring when you prescribe it in that setting versus having the patient come see me.” – Clinic-based pharmacist, FL

“I have to have a bond with them. I have to learn to understand them. They have to understand me too. That’s why I do everything through [Pharmacy Name], that team is great…I’m just afraid to go to any neighborhood drugstore pharmacy.” – Person with HIV on LA-ART, FL

High existing workload in clinics was a negative factor that could be an incentive for transitioning LA-ART administration from clinics to pharmacies.

“I have a list of 50 patients that are waiting to start Cabenuva that we can’t because we don’t have the capacity in our clinic. Yeah, so that would alleviate it and would allow us to provide that service for them.” – Specialty pharmacist, FL

Most of the perceived barriers shared by study participants related to space and privacy, pharmacy staffing, training, and reimbursement. Some pharmacists shared specific details regarding business operations that could make providing LA-ART services challenging and some acknowledged the increased difficulty of administering a ventrogluteal injection versus a routine vaccine provided intramuscularly in the deltoid muscle.

“I mean, just thinking of the pharmacy across the street, [they] don’t have space where patients can be vaccinated that they could lower their pants. I think operationally, it’s not necessarily set up to do in the pharmacy.” – Clinic-based pharmacist, FL

“Pharmacies, we are always experiencing staffing issues. That’s also taking a toll on us. They are working a little bit extra. I would say a little bit stressful, yes.” - Specialty pharmacist/Manager, TX

“Our business model …you sometimes will get sideways with the wholesaler if you order too many expensive brands because they want you to hit a certain percentage with the generics. So now you have a challenge because, again, you’re disincentivized to order brand name medications because you have a contract or an agreement in place with your wholesaler. So if you are a pharmacy that does a lot of HIV medications and you spent $100,000 a month just on these injectables, I mean that’s going to be very difficult to achieve your generic compliance rate.” – Community pharmacist, AL

### Perspectives of community pharmacies administering LA-ART

Three pharmacists represented two pharmacy locations that were administering LA-ART. These participants programs addressed barriers raised by others by expanding space, increasing staffing, obtaining clinic licenses to gain access to LA-ART, and diligently working on reimbursement, but these actions were not without challenges.

I think it’s important in the community setting where I own the pharmacy, we make the decisions locally, we determine our staffing level, right? As opposed to like working at [pharmacy], all those places I worked in the past, where your staffing model is determined by corporate. – Community pharmacist/Owner, CA

Sometimes we get paid, sometimes we don’t. But one way or the other, I give it to them and they get their medication, and they’re happy for it. Even if I’m not getting paid for it, at least I’m not losing money on the cost of the drug. And then, of course, [insurance] pays for it. It doesn’t pay amazingly, but $10 is $10. – Community pharmacist/Owner, CA

Inside of the pharmacy, we had a room that we used to see patients for. Because the volume of injections got too high, now we built another room in the past six months. – Specialty pharmacist/Manager, TX

Our leadership does track to see how we are trending and if we’re getting adequately reimbursed for it. The one thing we do not want is, we do not want our pharmacist stressing out like, “We need to do these many shots per month” or something like that. We just want to make sure that they have the time to do it and they have enough schedule. – Specialty pharmacist/Manager, TX

## Discussion

The United States has approximately 36,000 persons newly diagnosed with HIV annually [20]. Of those who are diagnosed and who receive care, 69% achieve an undetectable viral load within 6 months [21]. LA-ART could meet the needs of many people requiring treatment and health systems must prepare to accommodate a growing interest in these types of medications [22, 23]. Currently available LA-ART must be injected by a “healthcare provider”, which is broad terminology that should include pharmacists. Despite increasing demand and the challenges clinics are experiencing providing LA-ART, only a small handful of community pharmacies across the United States are performing LA-ART administration services [24–27]. To have a meaningful impact on access and equity, this differentiated care model would need to be scaled up beyond the currently existing small group.

One of the key findings of our study is that attitudes towards community pharmacy-administered LA-ART are favorable, and most felt that it would be an appropriate and helpful addition to options for administration. Attitudes about LA-ART administration in pharmacies amongst people with HIV were mixed due to their difficulty separating attitudes about administration location versus the medicines themselves. The congruence of the data provided an expanded understanding of the reasons people might support the development of these new programs; this could be helpful in determining how to best structure services to meet the identified gaps that make pharmacy administration desirable.

Although most participants believed developing and disseminating LA-ART administration services in pharmacies was feasible, another key contribution of our study is the documentation of important barriers, particularly those outlined by pharmacy staff members. Barriers were practical in nature and were common to developing any new healthcare service [28]. The essential questions raised included: (1) staffing and training (i.e., Who would be providing this service? How would they be trained? ); (2) location of the pharmacy, availability of space, and privacy (i.e., Where would the service be performed? How will privacy be ensured? ); and (3) sustainability of this service (i.e., How would the model for this service would be funded? ). Answering these critical questions will be required for successful program development program, and closely aligns with CFIR constructs of evaluating the inner setting (readiness for implementation, available resources) as well as specific aspects of administration in pharmacies (intervention characteristics: complexity, cost). The strongest facilitator for pharmacy-based LA-ART was relationships between persons with HIV and pharmacy staff, as well as between clinicians and pharmacy staff. This is consistent with published literature regarding pharmacy-based administration of long-acting antipsychotics, where patients are generally satisfied with services and where the described implementation facilitators and barriers are similar [29–31].

There are several critical next steps required for community pharmacies to adopt models of LA-ART administration more widely. Our study revealed some complexities involved in setting up pharmacy systems, clinic systems, and shared clinic-pharmacy systems required to successfully launch this type of service. Outlining universal workflow requirements, challenges, and identifying potential best practices would also be useful to help pharmacies initiate services. Pharmacies should take advantage of public familiarity and comfort with pharmacy-administered vaccines and they can leverage existing positive relationships they have built with clinic providers and patients [32]. This is a particularly poignant approach when developing services for persons with HIV and for communities at risk for HIV, given the potential presence of HIV-related stigma or trauma, and pressing needs for confidentiality and privacy. Having positive pharmacy experiences is a key factor for clinicians and persons with HIV to trust a newer model of LA-ART administration. Pharmacy-based LA-ART administration will require strong and committed partnerships between clinics, pharmacies, and persons with HIV or communities at risk for acquiring HIV given the longitudinal nature of the services. Clinic and pharmacy leadership will need to be willing to invest time and effort to explore this new model of care and must include persons with HIV or at risk for HIV in their planning processes.

Our study has limitations. This was an exploratory cross-sectional study that included self-reported data. Therefore, social desirability bias may have influenced the results. Despite our efforts to increase the generalizability of study findings by including a relatively large number of persons with HIV, pharmacists, and clinic staff from four different geographical locations in this mixed-methods study, the views and experiences of those in the study do not necessarily reflect those outside the catchment area. Our study only included persons able to speak English. Pharmacists in different states hold different scopes of practice, and this may influence what they believe is possible to achieve with a novel model of care.

## Conclusion

There is a unique opportunity to empower pharmacies to expand access to LA-ART for HIV treatment and prevention. Involving pharmacies in the administration of these medications may hold several positive benefits, including reducing clinic burden and increasing patient access. Our study found largely positive support for LA-ART administration within community pharmacies in three different stakeholder groups of persons with HIV, pharmacists, and clinic staff members. Since only a few existing pharmacies are currently administering LA-ART, disseminating information about practical strategies, ideal setups, potential barriers and best practices to overcome those barriers may help increase expansion of this important intervention.

## Supplementary Information

Supplementary Material 1.

## Data Availability

Data are available upon request to the study principal investigator. The team is in the process of placing de-identified data into a data repository at the University of California San Francisco.

## Abbreviations

LA-ART: Long-acting injectable antiretroviral therapy
HIV: Human immunodeficiency virus
IM: Intramuscularly administered
SC: Subcutaneously administered
CFIR: Consolidated Framework for Implementation Research
AIM: Acceptability of Intervention Measure
IAM: Intervention appropriateness measure
FIM: Feasibility of intervention measure
ANOVA: One-way analysis of variance
PrEP: Pre-exposure Prophylaxis
