# Supplementary material for: Preparing for pharmacy-based delivery of long-acting injectable antiretrovirals: a pre-implementation study
Source: BMC Health Serv Res. 2025 Jun 5;25:808. doi: 10.1186/s12913-025-12971-8 (PMC12139108; doi:10.1186/s12913-025-12971-8)
Supplement: Supplementary file 1 — Supplementary Material 1. [file 12913_2025_12971_MOESM1_ESM.docx]

Supplementary File 1: Interview Guides

[Semi-structured interview guide: Clinic staff members (v9/22) 2](#_Toc186450625)

[Semi-structured interview guide: Pharmacy staff members (v8/22) 5](#_Toc186450626)

[Semi-structured interview guide: Patients (v1/23) 10](#_Toc186450627)

# Semi-structured interview guide: Clinic staff members (including pharmacists who do not dispense medications who work inside of a clinic)

*Introduction: Thank you for taking the time to participate in this study. As you might already know, the FDA approved the first long-acting antiretroviral medications for the treatment of HIV in 2021. Today, I’ll be asking you a series of questions about your opinions, perceptions, and experiences with these long-acting injectable antiretroviral medications.*

*As mentioned in the consent form, I will make a sound recording of our conversation today to help the research team better remember what you said during the interview. Do you have any questions before we begin? Do I have your permission to begin recording? Let’s go ahead and begin.*

*<<BEGIN RECORDING>>*

1. **Please describe your clinic setting. What is your role there?**
2. **How do you obtain information about HIV, or more generally about your profession?**

- What professional networking do you engage in? Listservs? Local or national conferences? Trainings?

1. **Tell me some of the key things you’ve learned to-date about long-acting antiretroviral therapy.**
2. **Is your clinic currently offering injections of long-acting antiretroviral therapy to patients? Why or why not?**

- What influenced the decision to offer/not offer LAI-ART to your patient population?
- [If yes] What role do you play in the administration of LAI-ART?
- What have been some of the successes and challenges with implementing this service at your clinic?

*Right now, most patients receive their long-acting injectable ART at a clinic site. However, there is also the possibility of receiving these injections at alternative sites.*

1. **What do you think about having injections of long-acting antiretroviral therapy administered by/within community pharmacies?**

- What do you know about administering LAI-ART in pharmacies?
- Do you think this intervention will be effective in that setting? Why or why not?
- What kind of supporting evidence or proof is needed about the effectiveness of the intervention to get you or others on board?

1. **How does administration of long-acting injectable ART in pharmacies compare to other alternatives like getting the injections in the clinics, at home, or in other places?**

- What advantages does getting it in a pharmacy have compared to these other ways?
- What disadvantages does it have compared to these?

1. **How well does having LAI-ART administered/injected in the pharmacy fit with your existing work processes and practices in your setting?**

- What are likely issues or complications that may arise?
- **Can you describe how LAI-ART administration in pharmacies might/will be integrated into your current clinic processes or workflow? Walk me through the ideal process.** How will it interact or conflict with current programs or processes?
- Would this intervention replace or compliment your program or process? In what ways?

1. **Is there a strong need to administer LAI-ART within community pharmacies?**

- Why or why not? How will LAI-ART in pharmacies fill current gaps?
- What is the general level of receptivity in your organization to supporting LAI-ART administration in pharmacies? Why?

1. **How well do you think having LAI-ART administered by pharmacies will help meet the needs of individuals served by YOUR organization (clinic)?**

- In what ways would the intervention meet their needs? E.g. improved access to services? Reduced wait times?

1. **How do you think the patients will respond to having LAI-ART available to be given by pharmacies?**

- Have you ever talked with patients about their perceptions of getting LAI-ART in a pharmacy?
- What barriers will patients face to getting their LAI-ART in pharmacies?
- In what ways would the intervention meet their needs? E.g. improved access to services? Reduced wait times? Help with self-management? Reduced travel time and expense?

*Let’s talk about more of your opinions around having long-acting injectable antiretrovirals administered to patients by a community pharmacy*

1. **How confident are you that a pharmacy would be able to successfully administer LAI-ART?**

- What gives you that level of confidence (or lack of confidence)?

1. **How does a pharmacy’s infrastructure affect its ability to administer LAI-ART? What kinds of changes would need to be made for pharmacies to administer LAI-ART?**
2. **Thinking about your experiences with community pharmacies – the people there, their beliefs, values, assumptions -- How do you think pharmacy’s CULTURE will affect the implementation of administration of LAI-ART?**
3. **What level of endorsement or support have you seen or heard (if any) from pharmacy leaders about giving LAI-ART injections within a pharmacy?**

- Who are these leaders and how has this affected things so far?

1. **To what extent would implementing LAI-ART administration in a pharmacy provide an advantage to that specific pharmacy compared to others in your area?**
2. **What kind of financial or other incentives influence the decision to support administration of LAI-ART within pharmacies?**

- How will LAI-ART administration in pharmacies affect payment or revenue for YOUR organization?

1. **Who are the key influential individuals in your clinic who would need to get on board with LAI-ART injections being given in pharmacies?**

- What are these influential individuals saying (if anything) about administering LAI-ART in pharmacies?
- To what extent will they influence use of the pharmacy for LAI-ART? The success of the implementation in pharmacies?

1. **How would you want a pharmacy to communicate with you about the availability or logistics for this service of giving LAI-ART at their site?** What information would you want to be told?
2. **How would you or your colleagues communicate to your patients about this intervention?**
3. Thank you for answering all these questions. The discussion has been very helpful and interesting. Before we end, **is there anything about giving injections of long-acting antiretroviral therapy in pharmacies that I didn’t ask or that I left out, that you’d like to share with me today?**

*Thank you again for taking the time to participate in this study. <<STOP RECORDING>>.*

# Semi-structured interview guide: Pharmacy staff members

*Introduction: My name is XX and I am a researcher from the Department of Clinical Pharmacy at the UCSF School of Pharmacy. I’d like to thank you for taking the time to participate in this study. I wanted to follow up with you to see if you’ve read and understand the consent form, and ask if you have any questions about the study I can answer for you at this time?*

*As mentioned in the consent form, I will make a sound recording of our conversation today to help the research team better remember what you said during the interview. Do you have any questions before we begin? Do I have your permission to begin recording? Let’s go ahead and begin. We’ll start by talking more generally about your pharmacy.*

*<<BEGIN RECORDING>>*

1. **Please describe your pharmacy setting and your role. What types of services does your pharmacy provide for persons with HIV (or any other specific populations)?**
2. **Tell me about your working relationships with your colleagues. Your leaders/supervisors?**

- Do you meet (formally or informally) with a team of people? Who? How often?

1. **How would you describe the culture of your own setting/unit/pharmacy? Your (larger) organization?**

- Do you feel like the culture of your own unit is different from the overall organization? In what ways?

1. **How do you typically find out about new information, such as new initiatives, accomplishments, issues, new staff, staff departures?**

- When you need to get something done or to solve a problem, who are your "go-to" people?
- Can you describe a recent example?

1. **To what extent are new ideas embraced and used to make improvements in your organization?**

- Can you describe a recent example?
- Key players? Your involvement? Leader’s involvement? What factors made it successful/fail?

1. **To what extent do you feel like you can try new things to improve your work processes?**

- Do you feel like you have the time and energy to think about ways to improve things?
- Did you feel valued/respected by your supervisor for the role you played?

*Transition:* *As you might already know, the FDA approved the first long-acting antiretroviral medications for the treatment of HIV in 2021. Today, I’ll be asking you a series of questions about your opinions, perceptions, and experiences with these long-acting injectable antiretroviral medications.*

1. **Tell me some of the key things you’ve learned about long-acting injectable antiretrovirals OR about pharmacies administering them (meaning, actually injecting patients) in a pharmacy.**
2. **Is your pharmacy considering creating (or already running) a service for administering long-acting injections of antiretroviral therapy? IF NOT – how is your pharmacy involved (if at all) with the administration of LAI-ART?**

- (If implementing) At what stage of implementation is the intervention at in your organization?

*(NOTE: If needed, after q7 may share additional info for contextualizing discussion: These medications are given by injection once monthly or once every other month, by a healthcare provider. For currently approved medications, the injections are given in the gluteus muscle, ideally using a z-track method given larger volume (2 or 3 mL.)*

1. **Is there a strong need to administer LAI-ART within community pharmacies?**

- Why or why not? Do others see a need for the intervention?
- To what extent do current programs fail to meet existing needs?
- How will LAI-ART in pharmacies fill current gaps?

1. **How does injecting long-acting ART within pharmacies compare to other alternatives like getting the injection at the clinics, at home, or in other places?**

- Advantages? Disadvantages?

1. **How complicated (or not complicated) is it to give LAI-ART injections in community pharmacies?** *You can think about things like aspects of the actual administration: the duration, scope, intricacy and number of steps involved and whether this practice/service is aligned with things that are already done in a pharmacy. *Reminder: this ideally requires two, monthly or every-other-month ventro-gluteal injections (with a patient lying on their side for a z-track technique injection).*

- What kinds of changes or alterations would need to be made to long-acting injectable ART so it will work effectively in your setting?
- How would your pharmacy’s infrastructure facilitate/hinder implementation of long-acting ART injections?
- How will you work around structural challenges?
- Are changes in scope of practice needed? Changes in formal policies? Changes in information systems or electronic records systems? Other?
- Can you describe the process that will be needed to make these changes? What kind of approvals will be needed? Who will need to be involved?

1. **Can you describe how giving LAI-ART injections in a pharmacy might/will be integrated into current processes? Walk me through your ideal process.**

- How well does giving LAI-ART injections in the pharmacy fit with existing work processes and practices?
- How will it interact or conflict with current high priority programs or processes?

*Transition: With any new intervention, many people are often involved. These people may have differing approaches and opinions.*

1. **How would YOU feel about administering long-acting ART in your pharmacy?**

- Do you think this intervention will be/is effective in your setting?
- Would you have any feelings of anticipation? Stress? Enthusiasm? Why?

1. ***In healthcare, key stakeholders may include influential and well-respected clinicians, patients, managers, etc.* What do you think key stakeholders think of having LAI-ART administered by pharmacies?**

- Can you describe your working relationship with influential stakeholders such as key clinicians in your community?
- What kind of supporting evidence or proof is needed about the effectiveness of the intervention to get staff on board? Co-workers? Administrative leaders?

1. **How do you think your organization's culture (general beliefs, values, assumptions that people embrace) will affect the implementation of administration of LAI-ART in a pharmacy?**

- What is the general level of receptivity in your organization to implementing/supporting LAI-ART administration in pharmacies? Why?
- How well does administering LAI-ART in the pharmacy fit with your values and norms and the values and norms within the organization?

1. **What level of endorsement or support have you seen or heard from your pharmacy leaders about LAI-ART administration in the pharmacy, if ANY?**

- Who are these leaders and how has this affected things so far?
- What level of involvement has leadership at your organization had so far with the intervention?
- What kind of support or actions would you expect from your pharmacy leaders to help make implementation of LAI-ART administration in pharmacies successful?

1. **What local, state, or national performance measures, policies, regulations, or guidelines might influence the decision to administer LAI-ART in pharmacies?**

- What is the state of pharmacist provider status in your state?
- Are pharmacists allowed to administer injections in your state?

1. **What kind of financial or other incentives influence the decision to implement administration LAI-ART?**

- How will LAI-ART administration affect payment or revenue for your organization?
- What costs should be considered?

1. **To what extent would implementing LAI-ART administration in your pharmacy provide an advantage for your pharmacy compared to other pharmacies in your area?**

- Is there a competitive advantage?
- Is there something about the intervention that would bring more individuals into your organization, instead of another one in your area?

1. **Would you expect to have sufficient resources to administer LAI-ART in your pharmacy?**

- [If Yes] What resources are you counting on or would like to receive?
- [If no] What resources will not be available?
- Who will be involved in helping you get what is needed?

1. **What supports, such as online resources, marketing materials, or a toolkit, are available/should be made available to help implement LAI-ART administration in pharmacies?**

- How do you access these materials?
- What kind of training would be necessary for you? For colleagues?
- What kind of information exchange do you have with others outside your setting, either related to HIV, or more generally about your profession?
- What professional networking do you engage in? Listservs? Local or national conferences? Trainings?

1. **Have you talked with patients about their perceptions of getting LAI-ART in a pharmacy? How do you think the patients served by your organization will respond to having LAI-ART available to be administered in pharmacies?**
2. **How well do you think administering LAI-ART in pharmacies will help meet the needs of your patients?**

- In what ways would the intervention meet their needs? E.g. improved access to services? Reduced wait times? Help with self-management? Reduced travel time and expense? What barriers will patients face to getting their LAI-ART in pharmacies?

IF NOT CURRENTLY ADMINISTERING LAI-ART IN PHARMACY AND NOT CLOSE TO IMPLEMENTATION, read and end with q24: *Thank you for answering all of those questions. We’ve covered a lot!*

1. **Is there anything about giving injections of long-acting antiretroviral therapy in pharmacies that I didn’t ask or that I left out, that you’d like to share with me today?**

IF CLOSE TO IMPLEMENTATION or ALREADY IMPLEMENTED SERVICE, skip q24 and continue with q25

1. **You have either started administering long-acting injectable ART in your pharmacy or seem close to it. How was the decision made to start offering LAI-ART injections in the pharmacy?**
2. **Can you describe who was/is involved in the planning process?**
3. **In general, who are the key influential individuals who would need to get on board with administration of LAI-ART in the pharmacy?**

- What are these influential individuals saying about the intervention?

1. **Who will lead/is leading implementation of the intervention?**

- How did/will this person come into this role? Appointed? Volunteered? Voluntold?
- What attributes or qualities does this person have that makes them an effective leader of this implementation? What attributes or qualities does this person lack?
- Does this person have sufficient authority to do what is necessary to implement the intervention?

1. **Other than the formal implementation leader, are there people in your organization who are likely to champion administering LAI-ART in pharmacies?**

- Were they formally appointed in this position, or is it an informal role?
- What position do these champions hold in your organization?
- How do you think they will help with implementation?
- Can you describe people's perception of this champion/individual?
- To what extent do you respect the opinions and actions of the champion?
- What kinds of behaviors or actions do you think this individual/champion will exhibit?

1. **Would/will someone (or a team) outside of your pharmacy be helping you implement administration of LAI-ART injections in your pharmacy?**

- Can you describe this person/group? How did they get involved?
- What is their role? What kind of activities will they be doing?
- How helpful do you think he/she/they will be? In what ways?

1. **How will you or your colleagues communicate to the individuals that are served by your organization about the new service of getting LAI-ART injections by the pharmacist?**
2. **(Only if implemented) Has the intervention been implemented according to the implementation plan?**

- Why or why not?
- Which measures will you track? How will you track them? How will this information be used?

1. **To what extent has your pharmacy set goals around implementing giving long-acting ART injections in the pharmacy?**

- What are the goals? How will goals be communicated in the organization? To whom will they be communicated?

1. **Will you receive feedback reports about the implementation or the intervention itself?**

- What will they look like? Content, mode, form?
- How helpful do you think they will be?
- How often will you get them? Where will they come from?
- Who is designing them?

1. **How will you assess progress towards implementation or intervention goals?**

- Will feedback be elicited from staff? From the individuals served by your organization? [If yes] What kind of feedback?

1. Thank you for answering all these questions. The discussion has been very helpful and interesting. Before we end, **is there anything about giving injections of long-acting antiretroviral therapy in pharmacies that I didn’t ask or that I left out, that you’d like to share with me today?**

*Thank you again for taking the time to participate in this study. <<STOP RECORDING>>.*

# Semi-structured interview guide: Patients

Introduction*: Thank you for taking the time to participate in this study. As mentioned in the consent form, I will make a sound recording of our conversation today to help the research team better remember what you said during the interview. Do you have any questions before we begin? Do I have your permission to begin recording? Let’s go ahead and begin.*

*As you might already know, the FDA approved the first long-acting antiretroviral medications for the treatment of HIV in 2021. Today, I’ll be asking you a series of questions about your opinions, perceptions, and experiences with these long-acting injectable antiretroviral medications.*

*<<BEGIN RECORDING>>*

1. **Tell me your thoughts on LAI-ART. What have you heard about it? Is this something you would consider for yourself?**

*NOTE: If needed, after the answer to the question provide the following info about medications: “For the currently available long acting injectable HIV medicines, it requires two separate shots, given to you in your bottom (rear end) either once a month or once every two months. This may change as other long-acting HIV medicines become available.”*

*Right now, most patients receive their long-acting injectable ART medicines at a clinic, with shots given by a nurse. However, there is also the possibility of receiving these injections at different locations.*

1. **What has been your experience with your local pharmacy?**

- What services have you received there?
- What have been your experiences with the staff there?
- Describe your local pharmacy. What is it like?

1. **<<Even if you don’t want LAI-ART for yourself>>, [W]hat do you think about having LAI-ART injected by a pharmacist in a community pharmacy?**

- What do you know about getting LAI-ART in a pharmacy?
- What have you seen or heard (if anything) about getting LAI-ART injections in pharmacies?

1. **How does injection of long-acting injectable ART in a PHARMACY compare to other alternatives like getting the injection in a clinic, at home, or other places?**

- What advantages does getting it in a pharmacy have compared to these other ways? What disadvantages does it have compared to these?
- What barriers might you (or other patients) face trying to getting an LAI-ART medicine injection in a pharmacy?

1. ***How do you think pharmacy’s culture (the beliefs, values, and assumptions of people working in a pharmacy) affects the possibility of giving LAI-ART injections inside a pharmacy?***
2. **How well is YOUR pharmacy suited to give injections of LAI-ART?**

- What about it might help? What might stand in the way of pharmacies giving LAI-ART?
- What kinds of changes would need to be made for pharmacies to administer LAI-ART?

1. **How complicated would it be to have a pharmacist give you a LAI-ART injection in a community pharmacy?**

- Do you think getting a long acting injectable for HIV would be effective in that setting? Why or why not?

1. **How confident are you that a pharmacy would be able to successfully do injections of LAI-ART?**

- What gives you that level of confidence (or lack of confidence)?
- How would YOU respond to your pharmacy saying they would be able to inject LAI-ART for you?

1. **Is there a strong NEED to administer LAI-ART within community pharmacies?**

- What do you know about/think about existing programs/practices/processes to receive LAI-ART?
- To what extent do current programs fail to meet existing needs? How will LAI-ART in pharmacies fill current gaps?

1. **How will having LAI-ART injections in pharmacies help meet the needs of people with HIV?**

- In what ways would it meet their needs? E.g. improved access to services? Reduced wait times? Help with self-management? Reduced travel time and expense?

1. **If a pharmacy were to have LAI-ART injections available as a service, would that pharmacy have an advantage over others in your area?**
2. **The most common scenario right now is that a clinician (your doctor) prescribes the long-acting injectable HIV medicine and a nurse gives it to you. If pharmacies were able to give injections, how should the system work? Walk me through your ideal scenario.**

- How should they communicate with the provider?
- Who should be responsible for what?

1. **How would you want pharmacies to communicate with you about the availability or logistics for this service?**

- What information would you want to be told? What kind of information would you need/should be given about having LAI-ART given to you in a pharmacy?
- How would you want your clinic to communicate with you about the availability or logistics around getting a long-acting injectable ART at a pharmacy?

1. ***Who are the key influential persons who would need to get on board with pharmacies giving injections of LAI-ART?***

- *What are influential persons saying about the intervention?*
- *To what extent will they influence others' use of the intervention? The success of the implementation?*

1. **What advice would you give a pharmacy that wanted to start giving injections of LAI-ART to patients?**
2. **This information has been extremely interesting and informative. Is there anything else you’d like to share about having LAI-ART injections given in pharmacies that I did NOT ask you?**

*Thank you again for taking the time to participate in this study. <<STOP RECORDING>>.*
